# Experimental Comparison of Elastomeric Materials for Hydraulic Seal Durability Under Reciprocating Conditions

**DOI:** 10.3390/polym17233198

**Published:** 2025-11-30

**Authors:** Vishal Kumar, Muthu Elen

**Affiliations:** Energy and Environment Directorate, Pacific Northwest National Laboratory, Richland, WA 99354, USA; vishal.kumar@pnnl.gov

**Keywords:** Duralast, EPDM seals, wear rate, Archard model, seal durability

## Abstract

Wave Energy Converters (WECs) depend on hydraulic Power Take-Off (PTO) systems in which elastomeric seals must withstand wear, fatigue, and corrosion under harsh marine loading. This study quantitatively compares two commercial polyurethane seals (E1-E2) with custom-compounded Ethylene propylene diene monomer rubber (EPDM) formulations (E3–E5) using reciprocating wear tests (ASTM G133) at 3–10 N and 10–30 mm/s. It is noted that all experiments were conducted under dry conditions at room temperature as a baseline assessment, and the findings provide foundational insight prior to considering lubrication, hydraulic fluid effects, and marine environmental conditions relevant to WEC operation. Coefficient of friction (COF), specific wear rate, and worn-surface morphology were assessed to determine material durability. The commercial thermoplastic polyurethane (TPU) grades exhibited high hardness (93–94 Shore A), low wear rates (2.29–1.93 × 10^−4^ mm^3^/Nm), and shallow wear scars (≤380 µm). Carbon-black-reinforced EPDM (E3) produced the lowest wear rate among all samples (1.45 × 10^−4^ mm^3^ N^−1^ m^−1^) and the longest predicted service life (6.2 years), whereas silica-filled and plasticized EPDMs (E4, E5) showed higher wear (2.44–2.88 × 10^−4^ mm^3^/Nm) and broader deformation zones. Archard-based lifetime estimates at 10 N and 30 mm/s ranged from 3.1 to 6.2 years across materials. These results demonstrate that optimized EPDM formulations can serve as cost-effective alternatives to commercial TPUs for medium-load hydraulic sealing applications while providing a quantitative basis for material selection and life prediction.

## 1. Introduction

Wave energy is a promising renewable resource that can generate clean electricity by harnessing the kinetic energy of ocean waves through Wave Energy Converters (WECs). Hence, the marine energy systems must have lower operating costs, improve reliability, and advance technologies such as hydraulic Power Take-Off (PTO) systems, which convert wave motion into electricity. A critical component of these hydraulic PTO systems is the hydraulic seal, which prevents hydraulic fluid leakage and ensures watertight operation. However, seal degradation due to fatigue, corrosion, and sediment abrasion remains a major challenge, limiting maintenance-free operation and system reliability. Despite past studies on bearings and seal performance in related applications, limited research exists on the durability of piston seals in WEC PTO systems. Addressing this knowledge gap is essential for improving seal longevity, reducing operational costs, and promoting broader adoption of wave energy technologies. Despite this technological importance, existing literature provides limited continuity between seal function, degradation behavior, and their tribological origins.

Given the central role of seals in WEC performance, a deeper understanding of hydraulic seal behavior is critical not only for marine applications but also for broader hydraulic systems across multiple industries. Hydraulic seals are indispensable components in fluid power systems, ensuring confinement of pressurized fluids while preventing external contaminants. Their performance directly determines the reliability, efficiency, and service life of hydraulic machinery used in wave energy converter, aerospace, automotive, mining, and heavy engineering sectors. Seal failure is widely recognized as one of the leading causes of hydraulic system breakdown, with wear, frictional heating, and material degradation identified as the dominant mechanisms [1,2,3]. Among the various sealing applications, reciprocating rod and piston seals are particularly prone to failure due to cyclic stress, varying lubrication regimes, and complex thermal-mechanical interactions [4]. Although conceptually simple, hydraulic seals present a tribologically challenging system. The contact zone involves elasto-hydrodynamic lubrication, viscoelastic deformation, surface roughness interactions, and transient thermal effects [5,6,7]. Minor deviations in contact behavior, such as variations in film thickness or the occurrence of stick–slip instabilities, may lead to leakage, vibration, or catastrophic wear [8,9]. As such, continuous efforts are made to optimize seal material formulations, geometrical profiles, and lubrication strategies. Building upon these reliability challenges, prior studies have explored material- and interface-level responses, yet the findings remain fragmented across different elastomer systems.

Elastomers such as nitrile rubber (NBR), hydrogenated nitrile rubber (HNBR), fluoro elastomers (FKM), and polyurethane are the most common dynamic sealing materials [10]. Their elasticity, chemical resistance, and adaptability make them attractive choices, but their tribological properties remain highly sensitive to operating environments. For example, polyurethane seals exhibit excellent load-bearing and wear resistance but are prone to thermo-mechanical degradation under insufficient lubrication, where frictional heat accelerates microcracking and softening [11]. Fluoro elastomers are valued for high-temperature resistance yet suffer severe abrasive wear when exposed to particulate contaminants [12]. Furthermore, aging phenomena such as thermal oxidation, swelling, and fatigue synergistically accelerate the wear process [13]. Although these studies provide useful material-specific insights, a direct comparison across commercial and newly compounded elastomers under controlled reciprocating conditions is still lacking.

In response, researchers have attempted to improve seal performance by incorporating reinforcing fillers, applying surface treatments, and developing hybrid materials [14,15]. Thermoplastic-based sealing materials such as polytetrafluoroethylene (PTFE) and its composites offer low friction and excellent chemical stability but often suffer from cold flow and poor resilience [16]. Despite these advances, no universal material exists that meets all hydraulic sealing demands, emphasizing the need for application-specific development. Tribological evaluation of seals is generally based on three interdependent metrics: coefficient of friction (COF), specific wear rate, and surface roughness evolution. Friction governs both energy efficiency and system dynamics, with stick–slip instabilities often responsible for noise and vibration [17,18]. Wear defines the functional lifetime of the seal, progressively altering geometry, pressure distribution, and leakage resistance [19]. Surface roughness, in turn, mediates lubrication states and smoother surfaces after running-in may lower friction but can promote adhesion, whereas rougher surfaces often exacerbate leakage [20]. This disconnect highlights the need for a systematic evaluation linking friction, wear, and morphology across materials exposed to identical operating parameters.

Experimental studies confirm that seal behavior is strongly dependent on process parameters such as contact pressure, sliding speed, stroke, and lubrication state [1,7,21]. Distinct differences between in-stroke and out-stroke friction have been attributed to asymmetric fluid film formation [1]. Under abrasive conditions, rubber/steel pairs undergo three-stage transitions of wear mechanisms governed by particle fragmentation and movement [12]. Thermal effects further complicate the system as frictional heating alters molecular bonding within elastomers, mechanical properties reduce, and wear accelerates [11]. While many investigations have examined commercial sealing materials, comparative studies that integrate both industrial-grade seals and newly compounded elastomers remain limited. Moreover, most prior work has emphasized either friction or wear in isolation, rather than considering COF, specific wear, and surface morphology simultaneously across multiple process parameters. Mechanism-based interpretations linking experimental findings to lubrication and wear theory are also relatively scarce. Addressing this gap requires a unified experimental framework capable of comparing commercial and custom-compounded seals on the same tribological basis.

The focus of this research work is to evaluate the friction and wear of the two commercially available seal materials and compare them with the laboratory-compounded elastomers in a reciprocating wear test rig. The experiment measured specific wear rates, COF, and surface profile under a range of process parameters to capture both steady-state and transitional behaviors. By comparing commercial and compounded materials, the study seeks to elucidate performance trade-offs and to interpret wear and frictional responses in the context of existing theoretical models. The results contribute to material selection guidelines and offer a framework for developing customized compounded elastomers for demanding hydraulic sealing applications.

## 2. Materials and Methods

### 2.1. Materials

Two commercial polyurethane-based sealing materials were procured from American High-Performance Seals (AHP Seals, Oakdale, PA, USA). Duralast 4203 (E1) and Duralast 4758 (E2) are two of them. Duralast 4203 is a thermoplastic polyurethane (TPU) that has high resistance to wear and works with a wide range of hydraulic fluids. It stays stable in water up to 90 °C and meets FDA standards [19,20]. Duralast 4758 is a polyurethane that does not need to be lubricated and can withstand wear. It is meant to be used in situations where lubrication is not always available or is mixed. It keeps wear performance good while lowering stick slip and frictional heating [20,21]. The material compositions and physical properties were verified from the manufacturer’s technical datasheets to ensure data accuracy and traceability.

### 2.2. Compounding of EPDM Seal Materials

Using EPDM (Esprene 505) as the base polymer, four elastomer compounds were made concurrently in-house. Stearic acid, zinc oxide, mercaptobenzothiazole disulfide (MBTS, primary accelerator), tetramethylthiuram disulfide (TMTD, secondary accelerator), and sulfur were among the common curing ingredients found in the compounds, which were designated E3–E6. Carbon black and/or precipitated silica (Nipsil VN3) were used to vary the reinforcement. Dioctyl sebacate (DOS) was used as a plasticizer in one formulation. Carbon black was used to reinforce E3, silica was used for E4, a mixed filler system and plasticizer was used for E5. These differences made it possible to systematically investigate how filler and plasticization affect the tribological performance of seals made of EPDM. The composition of the compounded seal material is listed in Table 1. Each batch was compounded using a Thermo-Fisher Rheomix 600 internal mixer equipped with Banbury-style rotors. Mixing was conducted at a rotor speed of 40 rpm under temperature-controlled conditions maintained at 60 °C. Ingredients were introduced sequentially in the order listed in Table 1, added incrementally to ensure uniform dispersion and to prevent overloading. Throughout the mixing process, rotor torque was continuously monitored to assess the viscosity and dispersion behavior of the compound. After 15 min of mixing, the compounded materials were processed through a two-roll mill to achieve uniform sheet thickness and enhance filler distribution. Vulcanization was carried out in a 6 × 6 inch cavity mold with a 2 mm depth, following the procedure outlined in ASTM D3182. Curing was performed at 150 °C for 60 min to ensure complete crosslinking. In order to ensure comparability across all tested samples, the manufacturer directly supplied the commercial materials as circular specimens that were fabricated to the same dimensions as required by the experiment. All ingredient masses listed in Table 1 were recorded during formulation using a laboratory precision balance (±0.001 g). The compounding process strictly followed standard rubber-mixing protocols, and rheometric curves were used to determine optimal curing conditions, ensuring reproducibility of the material compositions.

### 2.3. Shore a Hardness

The Shore A hardness of both the commercially available seal materials and the compounded seal material was evaluated as per the ASTM D2240 (Standard) [16]. Test Method for Rubber Property-Durometer Hardness). Measurements were conducted at ambient temperatures on the flat surfaces of the molded specimens. Six measurements were obtained at various points on each material sample to reduce local variability. The average Shore A value was recorded as indicative of hardness. Each indentation was done at a minimum of 6 mm apart to prevent interaction between test spots, and the dwell period was 1 s for reading as stipulated by the standard.

### 2.4. Reciprocating Wear Test

**Figure 1:**
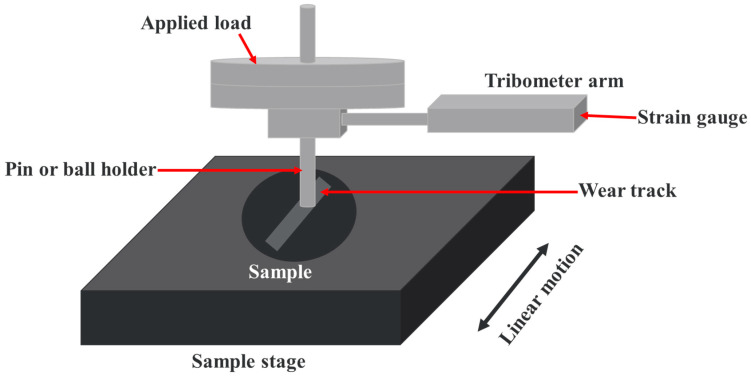
Schematic representation of reciprocating wear test.

Reciprocating wear tests were carried out using a CSM benchtop tribology tester (Anton Paar, Baden, Switzerland) operated in reciprocating mode, which is shown in Figure 1. The instrument employs dual friction-force sensors and a symmetrical elastic measuring arm, which minimizes thermal drift during extended runs. Hardware-corrected friction data were recorded continuously and stored. All the tests were conducted as per the ASTM G133 standards. Circular elastomer specimens were mounted in the sample holder and tested against a polished AISI 1045 steel ball (hardness ~50 HRC, Ra ≈ 0.05 µm). All experiments were performed under dry sliding at room temperature (25 ± 2 °C). The operating parameters used are listed in Table 2. All tribological tests in this study were conducted under dry conditions at room temperature against a steel counterface to establish a controlled baseline for comparing elastomeric materials. These conditions intentionally isolate material-dependent mechanisms prior to incorporating lubrication, hydraulic fluids, or marine-specific environmental factors present in WEC applications. Each condition was repeated three times to ensure reproducibility. Counter surface was cleaned with acetone before each run. The properties of counter surface and the seal materials are listed in Table 3.

Mass of the sample was measured before and after testing using a precision balance (±0.1 mg). Wear volume was calculated from mass loss and material density, and the specific wear rate was calculated. Worn tracks were additionally examined by optical profilometry for selected samples. Surface topography of the worn surface of the seal materials was characterized using a Keyence optical profilometer (VR-6100). Depth profiles and surface maps were obtained. The scanned area was 10 mm × 8 mm with a lateral resolution of 1.0–2.0 µm and a vertical resolution of 0.5 µm. A 20× objective lens was used, and surface data were processed with standard Gaussian filtering (cutoff 0.8 mm) and noise-reduction smoothing applied uniformly across all samples. These settings ensured reproducible measurements of wear-track width, depth, and morphology.

### 2.5. Statistical Analysis-2-Way ANOVA

A two-way analysis of variance (ANOVA) was performed to assess the impact of test parameters on the observed tribological responses, with normal load and sliding speed as independent variables and the coefficient of friction (COF) and specific wear rate as dependent variables. The investigation was conducted individually for each seal material (E1–E5) to identify the significant differences between load and sliding speed.

### 2.6. Lifetime Estimation of Seals Tested at Highest Load and Speed

The quantitative wear life of each seal material at highest load and speed was determined utilizing the classical Archard wear model [22] (Equation (1)), which correlates wear volume with applied load, sliding distance, and material wear rate. The corresponding lifetime to reach limiting wear volume (V_limit_) is determined by Equation (2).

V = kLs(1)

(2) $$T=\frac{V_{limit}}{kLv}$$

where V—wear volume (mm^3^); k—specific wear rate (mm^3^/Nm); L—applied normal load (N); s—sliding distance (m); and v—sliding velocity (m/s).

In the present investigation, the limiting wear volume is set at 850 mm^3^, which approximates 90% of the material volume of a sample with a diameter of 20 mm and a height of 3 mm (total volume ≈ 942 mm^3^). This threshold was chosen as the failure criterion beyond which dimensional stability and sealing efficiency would be lost. This threshold approximates the geometric point at which leakage and extrusion become unavoidable. Using a more conservative limit (e.g., 60–70%) would shorten the predicted lifetime proportionally. All tests were performed under a consistent load of 10 N and a sliding velocity of 30 mm/s.

## 3. Result and Discussion

### 3.1. Shore a Hardness of Seal Materials

**Figure 2:**
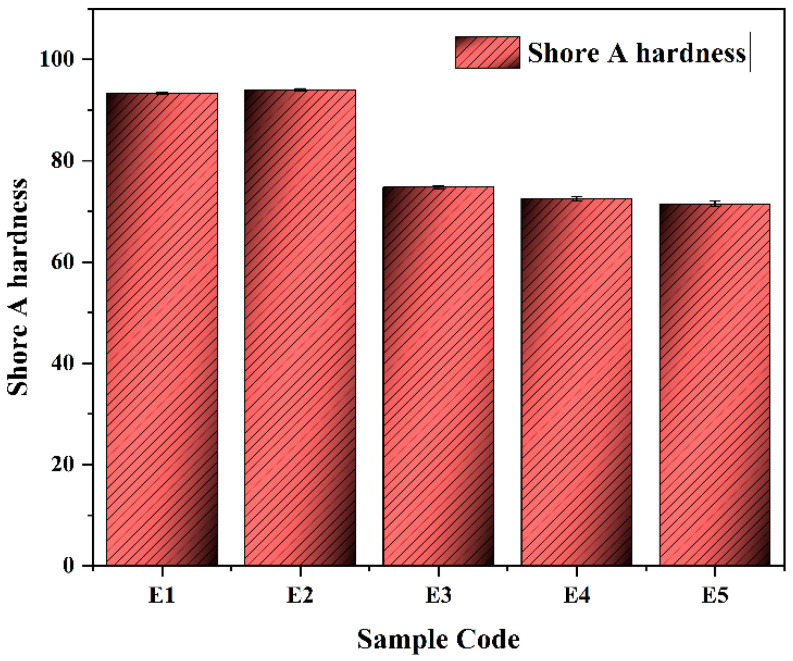
Shore A hardness of the seal materials.

The Shore A hardness of the compounded and commercial seal materials is displayed in Figure 2. Due to their high stiffness and dense cross-linked polyurethane structure, the commercial polyurethane-based seals (E1 and E2) had the highest hardness values of 93.3 and 94, respectively. These degrees of hardness are typical of thermoplastic polyurethane (TPU) formulations intended for hydraulic sealing at high pressures, when resistance to extrusion and dimensional stability are crucial. The hardnesses of E3, E4, and E5 are 74.8, 72.5, and 71.5, respectively; for compounded EPDM-based elastomers, on the other hand, they were much lower. These values result in a hardness reduction of roughly 20.4% (E3), 22.9% (E4), and 23.9% (E5) in comparison to the commercial seal material (E2 = 94). The reduction is ascribed to the impact of the plasticizing and reinforcing systems as well as the intrinsically lower modulus of the EPDM matrix. Among the EPDM compounds, Sample E3, which contained carbon black, had the maximum hardness because of its higher crosslink density and strong filler–rubber interfacial bonding [23]. On the other hand, E4 and E5, which were mostly supplemented with silica and modified with dioctyl sebacate (DOS) in the last case, displayed additional hardness decreases because of the plasticizer’s increased molecular mobility and decreased filler–matrix adhesion. The hardness trend shows that the commercial TPU seals are substantially stiffer than the formulations based on compounded EPDM. Hardness measured in this study is well within the ranges reported in earlier literature for similar sealing materials. Commercial TPU seals normally have Shore A hardness in the range of 90–95, as demonstrated in previous tribological studies on polyurethane-based hydraulic seals [11,19]. Similarly, EPDM-based seal compounds normally present hardness values between 65–75 Shore A, depending on the type of filler and crosslink density, as reported in several studies dealing with carbon-black- and silica-filled EPDM systems [22]. The measured hardness of our EPDM formulations (71.5–74.8 Shore A) is in good accordance with these reported values, confirming both the material integrity and the reliability of the mixing and curing procedure applied in this work. Tribological performance is anticipated to be impacted by this hardness gradient. Softer elastomers (E3–E5) may offer better conformability, damping, and sealing efficiency at lower contact pressures, whereas harder materials (E1, E2) generally offer higher resistance to wear and deformation [24].

### 3.2. Influence of Sliding Speed and Normal Load on Tribological Behavior of Seal Materials

**Figure 3:**
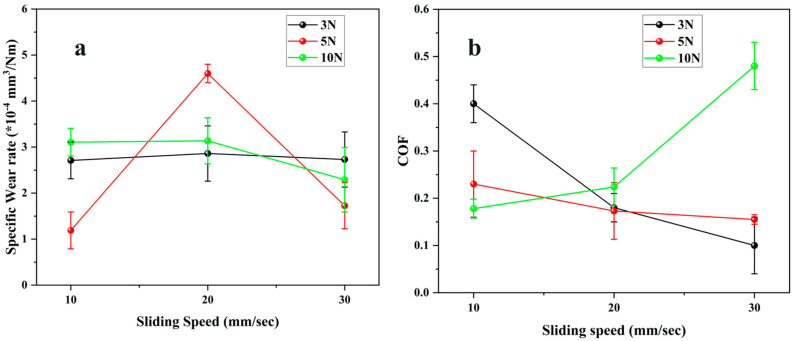
Tribological properties of E1 material (**a**) Specific Wear rate (**b**) Coefficient of friction.

Figure 3 depicts the change in specific wear rate and coefficient of friction (COF) for the commercial Duralast 4203 seal material (E1) over varying normal loads and sliding velocities. The trends indicate a distinct correlation between tribological performance and the operational parameters. The specific wear rate of E1 remained consistently low under all test conditions, proving the superior wear resistance of this thermoplastic polyurethane. Figure 3a illustrates that the wear rate ranged from 2.3 × 10^−4^ to 4.6 × 10^−4^ mm^3^/Nm, exhibiting only moderate sensitivity to both speed and load. At 3 N, the wear rate remained relatively constant (2.7–2.9 × 10^−4^ mm^3^/Nm) throughout all sliding velocities, signifying a steady micro-contact state and mild wear mostly governed by micro-abrasion [25]. At 5 N, a substantial rise in wear rate was noted at 20 mm/s, attaining 4.6 × 10^−4^ mm^3^/Nm, subsequently followed by a marked decline to 1.7 × 10^−4^ mm^3^/Nm at 30 mm/s. This temporary increase is probably attributable to localized surface softening and micro-tearing resulting from cyclic loading and inadequate cooling at intermediate velocities. The subsequent decrease indicates that increased velocity caused smoother sliding, less adhesion, and enhanced clearance of loose debris. Under the maximum load of 10 N, the wear rate was moderate (2.3–3.1 × 10^−4^ mm^3^/Nm), with a minor reduction at increased speeds. The stability under high load demonstrates Duralast 4203’s capacity to preserve structural integrity when subjected to compressive stress, attributed to its dense polyurethane matrix and superior elastic recovery [19,26]. The uniform wear resistance under all conditions highlights the material’s exceptional mechanical strength and surface durability. Figure 3b illustrates the coefficient of friction (COF) with respect to sliding speed and load. The coefficient of friction (COF) significantly decreased at 3 N, decreasing from 0.40 at 10 mm/s to 0.10 at 30 mm/s. This decrease is ascribed to surface polishing and the gradual growth of a thin transfer film that stabilizes the contact interface and lowers adhesive interactions of seal material. At 5 N, the coefficients of friction (COF) were generally lower (0.23–0.15) and exhibited a comparable decreasing trend, indicating the synergistic effects of an increased actual contact area and diminished asperity interlocking at moderate loads. In contrast, at 10 N, the coefficient of friction (COF) exhibited a substantial rise with velocity, rising from 0.18 at 10 mm/s to 0.48 at 30 mm/s. The increase indicates that at higher load and velocity, frictional heating caused viscoelastic softening and partial surface deterioration, hence augmenting hysteresis losses and adhesion. The increased tangential distortion and micro-shearing of the polyurethane surface certainly led to this rise in friction [26].

**Figure 4:**
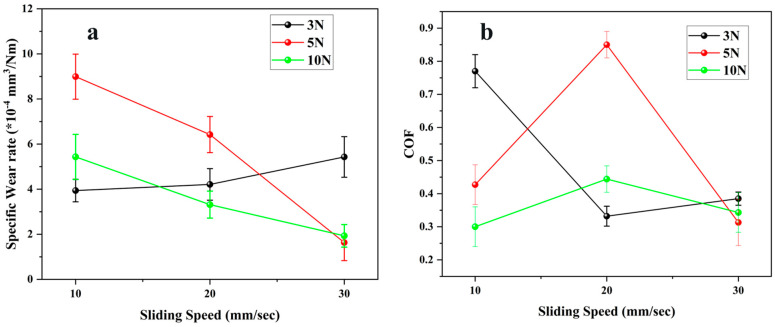
Tribological properties of E2 material (**a**) Specific Wear rate (**b**) Coefficient of friction.

Figure 4 shows the specific wear rate and coefficient of friction (COF) of Duralast 4758 (E2) as a function of sliding speed at normal loads of 3, 5, and 10 N. The wear rate of E2 seal material exhibited significant sensitivity to load and speed, ranging from 1.6 × 10^−4^ to 8.9 × 10^−4^ mm^3^/Nm, as illustrated in Figure 4a. At a minimal load of 3 N, the wear rate gradually increased with speed, rising from 3.94 × 10^−4^ mm^3^/Nm at 10 mm/s to 5.43 × 10^−4^ mm^3^/Nm at 30 mm/s, signifying a shift from moderate micro-abrasive wear to more pronounced adhesive or fatigue-assisted mechanisms at higher sliding velocities [25]. At 5 N, the wear rate showed an unusual trend, reaching a higher wear rate of 8.99 × 10^−4^ mm^3^/Nm at 10 mm/s, followed by a steep decline to 1.63 × 10^−4^ mm^3^/Nm at 30 mm/s. The substantial wear at low speeds and moderate loads is attributed to extended asperity contact and insufficient recovery of the elastomer surface between strokes, leading to micro-tearing [26]. As velocity escalated, the reduced contact duration and thermal softening facilitated smoother sliding and prevented material rupture. As velocity escalated, the wear rate consistently decreased from 5.43 × 10^−4^ to 1.93 × 10^−4^ mm^3^/Nm at the maximum load of 10 N. This outcome indicates that, despite increased contact pressure, the self-lubricating properties of Duralast 4758 enabled the development of a thin transfer layer, thereby preventing significant wear. The COF behavior, illustrated in Figure 4b, exhibited a more intricate relationship with load and velocity. At 3 N, the friction coefficient decreased significantly from 0.77 at 10 mm/s to 0.33 at 20 mm/s, thereafter, increasing somewhat to 0.39 at 30 mm/s. The initial drop relates to surface stabilization and partial polishing of the contact interface, whereas the later rise at elevated velocities is likely due to enhanced viscoelastic hysteresis and partial thermal softening of the near-surface layer. At 5 N, the coefficient of friction was 0.85 at 20 mm/s, indicating an unstable stick–slip phenomenon due to localized adhesion and interface thermal effects. At a higher speed of 30 mm/s, friction significantly reduced to 0.31, signifying a shift to a more consistent sliding condition with decreased adhesive effect. At 10 N, the coefficient of friction ranged from 0.30 to 0.44, indicating no significant instability. The moderate friction response under high load aligns with the improved self-lubricating properties of the seal material, which aids in mitigating direct asperity interlocking even at increased contact pressure [27,28].

**Figure 5:**
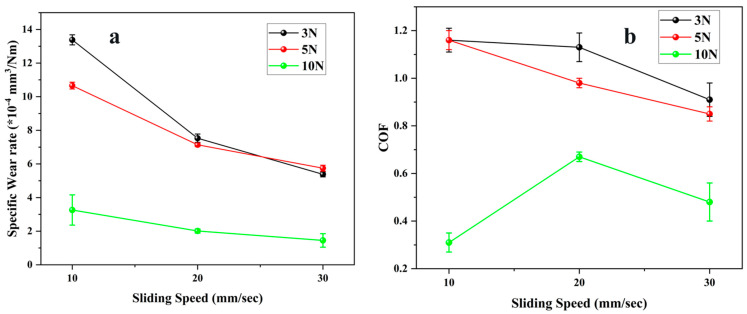
Tribological properties of E3 material (**a**) Specific Wear rate (**b**) Coefficient of friction.

Figure 5 depicts the relationship between specific wear rate and coefficient of friction (COF) for the E3 seal material as a function of sliding speed across various applied loads. The wear rate of the E3 seal material varied from 1.45 × 10^−4^ to 13.38 × 10^−4^ mm^3^/Nm, demonstrating a distinct inverse correlation with sliding speed and applied stress, as illustrated in Figure 5a. At 3 N, the specific wear rate decreased considerably from 13.38 × 10^−4^ mm^3^/Nm at 10 mm/s to 5.38 × 10^−4^ mm^3^/Nm at 30 mm/s, signifying a shift from severe to mild wear rate with increasing speed. This reduction can be due to reduced contact time and the potential development of a thin tribofilm on the counter pin that stabilized the interface at elevated sliding velocities [12]. At 5 N, a comparable decreasing trend was noted, with wear diminishing from 10.66 × 10^−4^ to 5.74 × 10^−4^ mm^3^/Nm as sliding velocity increased. The increased contact pressure under moderate load possibly increased micro-abrasive plowing at low speed, whereas greater speed facilitated less friction and the removal of loose worn debris, hence reducing additional material loss [29]. At the maximum load of 10 N, wear rate was relatively decreased throughout all velocities, declining from 3.26 × 10^−4^ at 10 mm/s to 1.45 × 10^−4^ mm^3^/Nm at 30 mm/s. The reduced wear under elevated loads indicates the onset of a mild wear regime, whereby the actual contact area has stabilized, and elastic deformation has absorbed most of the applied energy. The wear rate declines with increasing load and sliding speed, signifying that frictional heating may have helped localized softening, resulting in micro-contact and reduced material removal. The carbon black in E3 material likely improved stability by increasing crosslink density and the load-bearing capacity of the EPDM matrix. The COF data depicted in Figure 5b demonstrates a more intricate pattern under differing test settings. At 3 N, the coefficient of friction reduced from 1.16 at 10 mm/s to 0.91 at 30 mm/s, signifying a decrease in adhesive interaction and an improvement of the contact interface. At 5 N, a comparable pattern was noted, with the coefficient of friction decreasing from 1.16 to 0.85 as velocity increased. The elevated friction at lower speeds results from significant adhesive and viscoelastic hysteresis impacts on the elastomer surface [29]. The impacts reduce as the sliding velocity increases, and the contact becomes increasingly dynamic. At 10 N, the coefficient of friction (COF) initially increased from 0.31 at 10 mm/s to 0.67 at 20 mm/s, thereafter decreasing to 0.48 at 30 mm/s. The intermediate rise was due to the frictional heating and surface softness, which augment the actual contact area and adhesion. Conversely, at elevated speeds, a degree of self-polishing of the surface results in more stability during sliding. The irregularity observed across the 3, 5, 10 N is characteristic of elastomer tribology under dry reciprocating motion, where transfer-film formation, micro-scale thermal gradients, and dynamic filler–matrix interactions lead to abrupt changes in frictional response.

**Figure 6:**
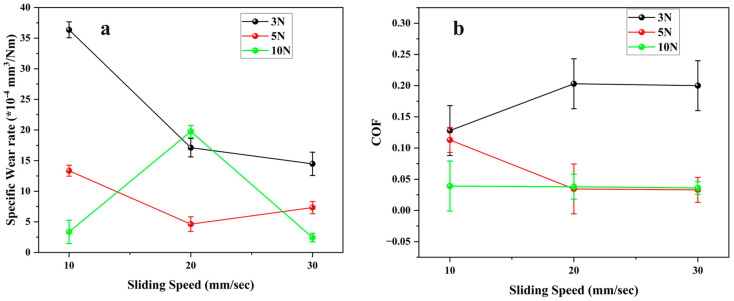
Tribological properties of E4 material (**a**) Specific Wear rate (**b**) Coefficient of friction.

Figure 6 displays the specific wear rate and coefficient of friction (COF) of the E4 seal material under varying sliding velocities and normal loads. At a load of 3 N, the wear rate decreased from 36 × 10^−4^ mm^3^/Nm at 10 mm/s to 14 × 10^−4^ mm^3^/Nm at 30 mm/s, exhibiting a reduction of almost 60%. The diminished wear rate with increased speed is due to decreased contact duration per stroke and lower asperity interlocking, which collectively enable a smoother contact interface and a uniform tribofilm, resulting in a moderate wear regime [17]. At 5 N, the wear rate initially dropped from 13 × 10^−4^ mm^3^/Nm to 4.6 × 10^−4^ mm^3^/Nm with increasing velocity, subsequently rising slightly to 7.3 × 10^−4^ mm^3^/Nm at 30 mm/s. At 10 N, the wear rate exhibited a distinct trend, initially rising from 3.4 × 10^−4^ mm^3^/Nm at 10 mm/s to 19 × 10^−4^ mm^3^/Nm at 20 mm/s, followed by a decrease to 2.4 × 10^−4^ mm^3^/Nm at 30 mm/s. This intermediate point signifies local fatigue or temporary micro-tearing resulting from cyclic stress concentration, which subsequently stabilizes as a tribofilm that develops at elevated speeds [30]. The wear response demonstrates that E4 shifts from a severe wear regime at low speed and low load to a stable mild-wear regime under high speed and high load. The silica filler increases wear resistance by increasing the modulus and thermal stability of the EPDM matrix, while also facilitating a more uniform distribution of applied stress across the contact region. The COF values for E4 were consistently low, ranging from 0.03 to 0.20, as illustrated in Figure 6b. At 3 N, friction exhibited a modest rise with speed, rising from 0.13 at 10 mm/s to 0.20 at 20 mm/s, and thereafter stabilizing at 0.20 at 30 mm/s. The marginal improvement is attributable to improved viscoelastic hysteresis and localized heating, which augment interfacial shear strength. At 5 N, the coefficient of friction diminished with increasing velocity, from 0.11 to 0.03, suggesting that elevated sliding speeds enhanced contact and mitigated adhesive friction. At 10 N, the coefficient of friction remained approximately constant at 0.04 ± 0.01, indicating that the surface attained a stable state with negligible additional deformation. The comprehensive tribological analysis indicates that the simultaneous increase in load and speed diminishes both wear and friction, resulting in a low-friction, low-wear regime at elevated loads and velocities. This trend results from the synergistic interaction between silica reinforcement and the elasticity of EPDM. The filler network bears the load, while the elastic matrix accommodates oscillating strain without significant surface failure [31]. The inclusion of finely scattered silica facilitates heat dissipation and limits extensive adhesive connections, thus ensuring consistent frictional performance.

**Figure 7:**
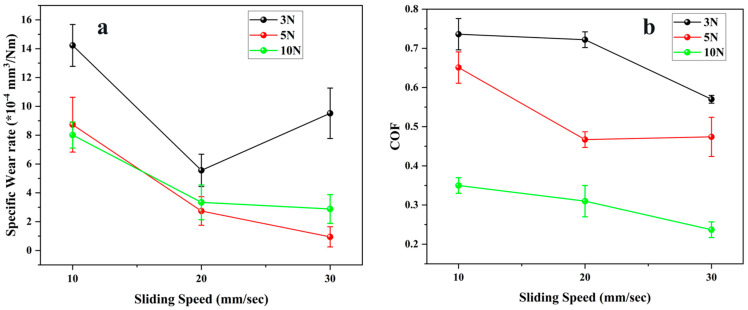
Tribological properties of E5 material (**a**) Specific Wear rate (**b**) Coefficient of friction.

Figure 7 illustrates the variations in specific wear rate and coefficient of friction (COF) of the tested polymeric seal material under different combinations of sliding speed and normal load. At the lowest load of 3 N, the specific wear rate (Figure 7a) decreases sharply from 1.42 × 10^−3^ mm^3^/Nm at 10 mm/s to 5.56 × 10^−4^ mm^3^/Nm at 20 mm/s, before rising again to 9.52 × 10^−4^ mm^3^/Nm at 30 mm/s. This behavior can be attributed to an initial reduction in the real contact area and adhesive of the polymer as speed increases, followed by mild thermal softening and unstable film formation at higher sliding velocities. At moderate and higher loads (5 N and 10 N), wear rate decreases consistently with increasing speed, attaining minimum values of 0.95 × 10^−4^ mm^3^/Nm and 2.88 × 10^−4^ mm^3^/Nm, respectively, at 30 mm/s. The reduction in wear at higher load and speed combinations is indicative of a transition toward a more stable tribo-film regime, wherein the elevated interfacial temperature enhances polymer softening and adhesion to the counter face, producing a self-protective layer that limits material removal and changes the contact from polymer-metal to polymer-polymer, reducing the wear rate [16,32].

A similar trend is reflected in the COF, which is displayed in Figure 7b. At 3 N, the COF maintains a relatively high value throughout the tested speed range, indicating a predominant contribution from adhesive and plowing of the polymer surface. Increasing the load to 5 N lowers the COF from 0.65 to 0.47 as the sliding speed rises to 20 mm/s, beyond which it stabilizes, likely due to the formation of a uniform transfer film that mitigates direct contact with the countersurface. The lowest COF values were recorded at 10 N, highlighting the beneficial effect of higher normal pressure in promoting real contact homogenization and transfer-film continuity, which together reduce interfacial shear stress, reducing the friction between the polymer surface and the metal counter surface [24]. It is important to note that elastomers do not exhibit linear scaling of friction or wear with load; instead, they transition between adhesive, abrasive, and hysteresis-dominated regimes as the interfacial temperature and real contact area fluctuate.

Although the results of this study demonstrate clear trends in friction, wear rate, and deformation under dry reciprocating contact, lubrication, hydraulic-fluid chemistry, temperature gradients, and hydrostatic pressure further complicate the tribological behavior of hydraulic seals in WEC systems. Pressurized hydraulic oil or water-based fluids reduce adhesive friction and suppress micro-abrasion by forming boundary or mixed lubrication films. Marine salinity and water ingress may accelerate hydrolysis in some polyurethane grades or modify filler-matrix interactions in EPDM-based compounds. Temperature changes affect viscoelastic response, while pressure affects real contact area and sealing stress. Therefore, whereas the dry-sliding ranking described here, E3 > E2 > E1 > E4 > E5, is indicative of low-lubrication and/or start-up conditions, its applicability under fully lubricated hydraulic operation may be quite different. The findings thus provide a baseline framework on which subsequent studies in fluid-immersed and marine environments can be built.

### 3.3. Depth-Profile Characterization of the Tested Seal Material Under High Load and High Speed

**Figure 8:**
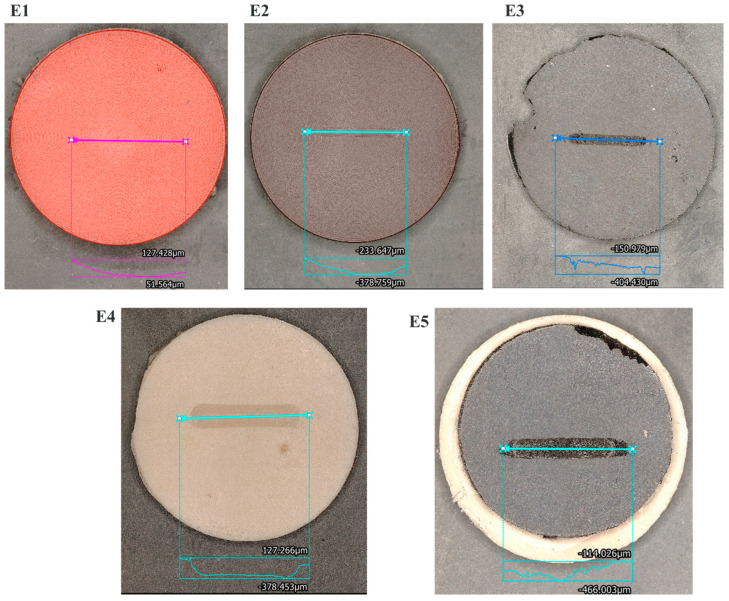
Depth profile of tested seal materials at higher load and speed.

Figure 8 displays the depth profile of all seal materials under the most severe test condition (10 N, 30 mm/s). This condition was selected to represent the fully developed wear state and to compare the steady-state morphology among the different formulations. The optical depth-profile analysis gives quantitative details about the surface deformation behavior and correlates directly with the measured wear-rate data. The commercial polyurethane seals (E1 and E2) exhibited the smallest track depth, approximately 50–380 µm, and relatively smooth edges, indicating limited abrasive removal and effective elastic recovery during reciprocating motion. The high cross-link density and hardness of these thermoplastic polyurethane matrices restricted surface penetration and promoted a stable contact interface [10,20]. Between the two grades, E2 shows a slightly deeper groove that is narrow, suggesting localized shear through its self-lubricating formulation rather than bulk material loss. The EPDM-based seal materials (E3–E5) showed more pronounced wear tracks, with depths ranging from 380 to 466 µm and larger deformation zones, reflecting their softer, energy-dissipative matrix. The E3 formulation (carbon-black-filled EPDM) showed a rougher and uneven wear scar, typical of micro-abrasive plowing and filler pull-out. In contrast, the silica-reinforced E4 and E5 samples exhibited more compacted wear zones, implying better filler–matrix reinforcement and improved interfacial stability [28]. Particularly, the E5 surface showed smoother curvature at the groove base and reduced waviness, which can be attributed to the presence of the dioctyl sebacate (DOS) plasticizer that enhanced flexibility and reduced crack propagation [33].

### 3.4. Wear Track Width Analysis of the Tested Seal Materials

**Figure 9:**
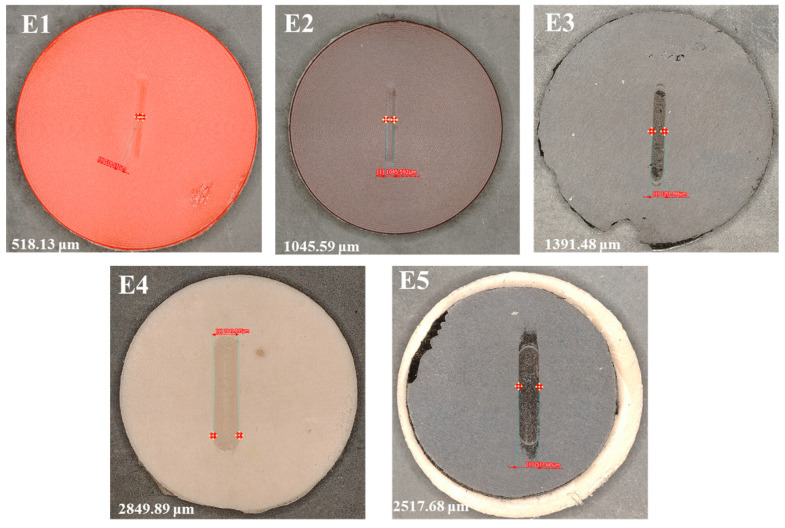
Wear track width analysis of tested seal materials at higher load and speed.

Figure 9 displays optical images of the worn surfaces for all seal materials (E1–E5) tested at a load of 10 N and a sliding speed of 30 mm/s. The measured wear track width values are inscribed on each image, indicating the degree of surface deformation and removal of material during steady-state reciprocating motion. The commercial polyurethane seals (E1 and E2) exhibited the smallest wear tracks among all investigated seal materials, measuring around 518 µm and 1046 µm, respectively. The shallow and narrow track shows enhanced elastic recovery and resistance to applied loads [30,33]. This behavior is characteristic of high-modulus polyurethane systems, wherein extensive crosslinking and elevated Shore A hardness reduce plowing and limit viscoelastic creep under cyclic loading. Among the two commercial grades, E2 exhibited a marginally broader track while preserving a smooth and uniform surface morphology, in accordance with its balanced formulation that integrates durability with constrained self-lubrication. Alternatively, the EPDM-based seals (E3–E5) display increasingly broader wear tracks, which relate to reduced hardness. The E3 sample (EPDM + carbon black) exhibited a width of about 1391 µm, indicating more lateral deformation and micro-abrasive plowing due to the hard filler agglomerates. The E4 specimen (EPDM + silica) exhibited the largest track (~2849 µm), signifying considerable surface softening and partial rupture of the elastomeric matrix. This phenomenon results from localized stress concentration around inadequately bound silica aggregates, facilitating fracture initiation and debris formation [34]. Surprisingly, the E5 formulation (EPDM + silica + DOS plasticizer) exhibited a reduced wear track (~2518 µm) compared to E4, despite its more pliable makeup. The use of dioctyl sebacate (DOS) enhanced molecular flexibility and stress relaxation, allowing the surface to deform uniformly instead of fracturing under cyclic load. The damping action governed by the plasticizer diminished the intensity of asperity cutting and restricted the spread of surface cracks [35], correlating with its reduced wear rate and friction coefficient.

The quantitative profilometry parameters listed in Table 4 support the surface features shown in Figure 8 and Figure 9 and help correlate the measured topography with the dominant wear mechanisms. TPU samples (E1 and E2) showed the shallowest grooves (50–380 µm) and lowest roughness values (Ra = 3.2–6.8 µm), reflecting mild micro-abrasion and partial elastic recovery typical for high-modulus polyurethanes. In contrast, significantly deeper tracks were produced by EPDM-based materials (410–466 µm), along with higher roughness, reflecting stronger contributions from micro-plowing, filler–matrix debonding, and viscoelastic deformation. For E3, the deepest valleys amount to 466 µm, while the highest Sa values correspond to 15.3 µm. This sharp, V-shaped grooving is typical of carbon-black-induced plowing. Carbon black forms stiff agglomerates that act as micro-cutting asperities, which promote abrasive penetration and filler pull-out. Indeed, Zhou et al. [7] have reported similar plowing behavior for carbon-black-filled EPDM under reciprocating motion. E4 showed steep-sided grooves and brittle ridge formation, consistent with silica-matrix debonding and crack initiation. Silica’s rigid, hydrophilic surface imparts higher stress concentration, leading to micro-crack propagation and fragmented debris. Literature on silica-filled EPDM similarly reports crack-dominated wear due to weak filler–rubber interfacial bonding [33]. The smoother groove base and lower roughness (Ra = 8.9 µm) of E5 are attributed to DOS plasticizer improving chain mobility and reducing local rigidity. Plasticizer-induced softening allows the matrix to redistribute stresses during reciprocating loading, limiting crack formation and producing broader but shallower deformation zones. This is in line with previous findings that plasticized EPDM displays improved damping and reduced brittle wear [30,31].

It is important to remember that the wear mechanisms found in this study are for dry, room-temperature sliding and will change when the WEC is fully lubricated. Hydraulic fluids typically reduce adhesive friction and suppress micro-abrasion by forming boundary or mixed lubrication films, while fluid chemistry and marine salinity may influence polymer–filler interactions and accelerate hydrolytic or oxidative pathways in some formulations. Temperature gradients and operating pressure also alter viscoelastic response and real contact area during reciprocating motion. Therefore, the material ranking reported here (E3 > E2 > E1 > E4 > E5) is most representative of start-up phases, boundary-lubrication regimes, transient low-oil conditions, or periods of wave-induced load fluctuation, where dry or near-dry contact is more likely. Under fully flooded hydraulic operation, the absolute wear rates may change, but the dry-sliding results provide a necessary baseline for understanding intrinsic material behavior.

### 3.5. Statistical Analysis 2-Way ANOVA

A two-way analysis of variance (ANOVA) was utilized to statistically investigate the effects of normal load and sliding speed on the tribological characteristics of seal materials, at a 0.05 level of significance. The calculated F-ratios for both parameters were compared with the appropriate critical values (F_0_._05_), and the relevance of each factor was determined by assessing whether the computed value exceeded the tabulated value. The analysis was conducted independently for each material, utilizing the average data from three experimental replicates of the coefficient of friction (COF) and specific wear rate (in Supplementary Materials).

For E1 (Duralast 4203), the computed COF were Fₗ (2,18) = 180.84 > 3.55 and Fₛ (2,18) = 93.35 > 3.55, demonstrating that both load and speed substantially influenced friction. The specific wear rate showed Fₗ (2,18) = 88.54 > 3.55 and Fₛ (2,18) = 148.30 > 3.55, indicating a large variation in wear rate with respect to operating parameters. Consequently, both load and sliding velocity significantly influenced the tribological behavior of E1.

For E2 (Duralast 4758), the F-values for the COF were Fₗ (2,18) = 240.10 > 3.55 and Fₛ (2,18) = 196.60 > 3.55, indicating a significant influence of friction on both load and speed. The wear rate exhibited a notable difference, with Fₗ (2,18) = 957.06 > 5.14 and Fₛ (2,18) = 1135.60 > 5.14, demonstrating that both parameters significantly affected the wear rate of the seal material.

The ANOVA results for E3 (carbon-black-filled EPDM) indicated Fₗ (2,18) = 355.20 > 3.55 and Fₛ (2,18) = 54.70 > 3.55 for COF, suggesting substantial variation in both parameters. The specific wear rate indicates that Fₗ (2,18) = 1280.00 > 5.14 and Fₛ (2,18) = 1180.00 > 5.14, demonstrating that load and speed significantly influenced wear intensity, largely attributable to the reinforcing effect of carbon black and its enhancement of load-bearing capacity.

Surprisingly, E4 (silica- and plasticizer-modified EPDM) exhibited no significant variation in the COF, with Fₗ (2,18) = 0.39 < 3.55 and Fₛ (2,18) = 0.39 < 3.55, indicating consistent frictional performance under all testing conditions. The specific wear rate exhibited significant variability, with Fₗ (2,18) = 612.40 > 5.14 and Fₛ (2,18) = 548.70 > 5.14, indicating that wear remained responsive to loading and speed despite consistent COF. This indicates that the silica-plasticizer mixture enhanced surface damping but failed to avert subsurface fatigue wear.

For E5 (unfilled EPDM reference), both the coefficient of friction and wear exhibited statistically significant influence on load and velocity. The computed COF were Fₗ (2,18) = 412.60 > 3.55 and Fₛ (2,18) = 266.20 > 3.55, while the wear values were Fₗ (2,18) = 889.10 > 5.14 and Fₛ (2,18) = 942.50 > 5.14, demonstrating that the unreinforced material exhibited the highest sensitivity to external mechanical forces. The computed F-values for nearly all materials, except for the COF of E4, are above their critical thresholds at a 95% confidence level, indicating a significant difference among the applied load and sliding speed levels. The findings indicate that both friction and wear are influenced by interrelated mechanical and dynamic factors rather than by a single parameter [36]. In materials comprising carbon black and polyurethane matrices, the interplay between load and speed generated significant synergistic effects attributable to thermal activation and interfacial densification. Conversely, the silica-modified EPDM exhibited a consistent COF yet demonstrated wear that was contingent upon applied stress. This indicates that internal viscoelastic losses constrained friction variation without diminishing surface fatigue. The ANOVA results quantitatively demonstrate that the tribological behavior of sealing materials is highly influenced by both load and sliding speed [37], providing quantitative evidence of their interconnected effects on frictional energy dissipation and wear mechanisms.

### 3.6. Lifetime Estimation of Seal Materials

Based on the experimentally determined wear rate, the estimated service lifetimes were calculated for each seal material (E1–E5) using the equations given in Section 2.6. The results are summarized in Table 5.

The predicted lifetime varied from about 3.1 years (E5) to 6.2 years (E3) under the specified operating conditions. Although showing comparable coefficients of friction, E3 had the lowest wear rate (1.45 × 10^−4^ mm^3^/Nm) and hence the longest operational lifespan. In contrast, E5 demonstrated a comparatively higher wear rate, signifying accelerated material loss and lower durability. The findings indicate that wear rate has a more significant impact on service life than the frictional coefficient within the examined parameter range. Even slight decreases in the specific wear rate considerably prolonged the anticipated operational lifespan, aligning with previous tribological models in which the wear rate dictates volumetric material loss for longer periods [22,38]. The tribological stability of E3 results from superior load-bearing film development and enhanced interfacial cohesion during repeated sliding, while E4 and E5 suffered early material degradation, probably due to localized micro-fatigue and matrix fragmentation [30]. These findings offer a practical quantitative approach for forecasting seal longevity or component replacement timelines based on empirical wear data.

Although the applied loads (3–10 N) and speeds (10–30 mm/s) represent laboratory-scale testing, the corresponding contact pressures (~0.3–1 MPa) replicate the near-surface stresses experienced at the rod–seal interface in operating hydraulic systems. Thus, the mechanisms observed, like adhesive transfer, micro-abrasion, viscoelastic deformation, and filler-matrix debonding, reflect the same degradation processes that occur under elevated industrial pressures. The specific wear rate, measured in mm^3^/Nm, is a normalized, scale-independent number that can be used to directly compare to real-world conditions using the Archard wear relation. To relate these estimates to realistic WEC operating conditions, the applied load (10 N) and sliding velocity (30 mm/s) represent a simplified upper-bound duty cycle for a point-absorber piston seal. Typical WEC systems operate at 0.2–1 Hz, corresponding to approximately 17,000–86,000 strokes per day. For a 20 mm stroke length, this value yields an approximate yearly sliding distance of 365 km. Thus, the Archard-based predictions correspond to the cumulative distance a WEC piston seal would experience over several years of operation, providing a reasonable first-order equivalence between laboratory measurements and field exposure. Industries can apply these experimentally derived coefficients in combination with actual load and stroke data to estimate seal life, define safe operational envelopes, and develop accelerated durability tests.

## 4. Conclusions

This research was performed to evaluate how load and sliding velocity govern the tribological behavior and service life of elastomeric seals commonly used in wave energy converters’ hydraulic piston system, thereby contributing to the marine energy developers’ mission to enhance component durability and energy efficiency in hydraulic and marine systems. These results represent a baseline dry-sliding assessment of seal materials, and the observed ranking is most applicable to low-lubrication, boundary-contact, or startup conditions rather than fully fluid-lubricated WEC operation. The tribological properties of commercially available thermoplastic polyurethane (TPU) and laboratory-compounded EPDM-based elastomeric seals have been investigated under reciprocating sliding conditions at varying loads and sliding velocities. Based on the present investigation and findings, the subsequent conclusions can be derived.

The Shore A hardness of the commercial Duralast seals (E1 and E2) was 93.3 and 94, respectively, demonstrating exceptional dimensional stability and load-bearing capacity. The EPDM-based compounds (E3–E5) exhibited reduced hardness values ranging from 74.8 to 71.5, reflecting a decrease of 20 to 24%. The specific wear rate at the maximum load and sliding velocity ranged from 1.45 × 10^−4^ mm^3^/Nm (E3) to 2.88 × 10^−4^ mm^3^/Nm (E5), but the coefficient of friction fluctuated between 0.036 (E4) and 0.480 (E1 and E3). The carbon-black-filled E3 exhibited the minimal wear rate and a consistent coefficient of friction, owing to enhanced crosslink density, uniform tribofilm formation and interfacial load transfer ability. The depth-profile analysis under higher test conditions (10 N, 30 mm/s) showed wear track depths of 50–380 µm for the polyurethane seals (E1, E2) and 380–466 µm for the compounded EPDMs (E3–E5). The shallow grooves in TPU grades show significant elastic recovery, whereas the deeper grooves in EPDM show enhanced viscoelastic deformation and filler detachment. Optical profilometry revealed wear track widths of 518 µm (E1), 1046 µm (E2), 1391 µm (E3), 2849 µm (E4), and 2518 µm (E5). The small scars in E1–E2 result from mild micro-abrasive wear, but the larger tracks in E4–E5 result from filler debonding and matrix fatigue. E5 exhibited a more uniform curvature due to tension relaxation mediated by DOS. The Archard model predicts a lifespan (V_limit_ = 850 mm^3^, L = 10 N, v = 0.03 m s^−1^), estimating operational durability between 3.12 years (E5) and 6.20 years (E3). A slight reduction in wear rate (~1 × 10^−4^ mm^3^/Nm) substantially doubled the anticipated service life, confirming that wear rate is the primary determinant of seal longevity over coefficient of friction (COF). The comprehensive rating for tribological performance was E3 > E2 > E1 > E4 > E5. The carbon-black-reinforced EPDM (E3) exhibited optimal amalgamation of wear resistance, friction stability, and anticipated lifespan (about 6 years). The reported lifetimes represent a baseline dry-sliding estimate, and while absolute values may shift with lubrication or a more conservative wear threshold, the relative ranking of materials remains unchanged due to consistent differences in wear-rate performance. The research suggests that optimized EPDM composites may compete with TPU seals in terms of durability, while providing enhanced damping and cost-effectiveness for reciprocating hydraulic applications. The insights from this study enable marine energy developers to optimize seal formulation, filler selection, and hardness for specific duty cycles, supporting predictive maintenance and extended service intervals. By coupling material-level wear data with system-level models, this work establishes a bridge between laboratory tribology and full-scale hydraulic performance, offering both a scientific framework and a practical design tool for marine energy applications focused on achieving long-life, low-maintenance, and energy-efficient sealing technologies.

## 5. Future Work

This study demonstrates the influence of load and sliding velocity on the wear and frictional properties of elastomeric hydraulic seals, providing critical information to designers of marine energy systems to improve the reliability and durability of fluid power systems in marine applications. Subsequent research will broaden this investigation to encompass water-lubricated and submerged operational environments, simulating the pressure and salinity conditions essential for wave energy converters and offshore hydraulic systems. Future research must focus on thermal aging and cyclic fatigue analysis of hydraulic seals to simulate extended deterioration in real operational conditions. Integrating experimentally derived wear data with system-level simulations and life prediction models can improve marine energy components’ reliability databases and help with establishing design standards for advanced hydraulic seals. The predictive lifespan models developed function as an analytical instrument for industry and national laboratories to improve seal material selection, establish operational limits, and reduce downtime in energy-harvesting devices.

## Figures and Tables

**Table 1:** Composition of the compounded EPDM seal materials.

Material (g)	Composition		
	E3	E4	E5
EPDM (Esprene 505)	46.54	47.28	40.22
Stearic acid	0.46	0.47	0.40
Zinc oxide	2.32	2.36	2.01
MBTS	0.69	0.71	0.60
TMTD	0.32	0.33	0.28
DOS	-	-	4.02
Carbon Black	11.63	-	8.44
Silica (Nipsil VN3)	-	14.18	10.05
Sulfur	0.69	0.71	0.60

**Table 2:** Process parameter used in reciprocating wear test.

Parameter	Value(s)
Load (N)	3, 5, 10
Sliding speed (mm/sec)	10, 20, 30
Stroke Length (mm)	20
Sliding duration (hours)	24

**Table 3:** Properties of counter surface and seal materials.

Properties	Seal Material					Counter Surface
	E1	E2	E3	E4	E4	AISI 1045 Steel Ball
**Density (g/cm^3^)**	1.18	1.20	1.09	1.11	1.08	-
**Diameter (mm)**	20	20	20	20	20	5
**Specimen thickness (mm)**	3	3	3	3	3	-
**Hardness**	93.3	94	74.8	72.5,	71.5	54 HRC
**Surface roughness (µm)**	-	-	-	-	-	0.05

**Table 4:** Profilometry values at 10 N and 30 mm/s.

Material	Max Depth (µm)	Ra (µm)	Sa (µm)
E1 (TPU 4203)	50	3.2	4.1
E2 (TPU 4758)	380	6.8	8.4
E3 (EPDM + Carbon Black)	466	12.5	15.3
E4 (EPDM + Silica)	442	10.8	13.6
E5 (EPDM + Silica + DOS)	410	8.9	11.4

**Table 5:** Estimated lifetime of seal materials.

Sample	COF	Wear Rate (mm^3^/Nm)	Estimated Lifetime (Years)
E1	0.480	2.29 × 10^−4^	3.93
E2	0.343	1.93 × 10^−4^	4.66
E3	0.480	1.45 × 10^−4^	6.20
E4	0.036	2.44 × 10^−4^	3.68
E5	0.237	2.88 × 10^−4^	3.12

## Data Availability

The raw data supporting the conclusions of this article will be made available by the authors on request.

## Supplementary Materials

The following supporting information can be downloaded at https://www.mdpi.com/article/10.3390/polym17233198/s1.
