# Supplementary material for: Experimental Comparison of Elastomeric Materials for Hydraulic Seal Durability Under Reciprocating Conditions
Source: Polymers (Basel). 2025 Nov 30;17(23):3198. doi: 10.3390/polym17233198 (PMC12694298; doi:10.3390/polym17233198)
Supplement: Supplementary file 1 [file polymers-17-03198-s001.zip › polymers-4015118-supplementary.pdf]

Experimental Comparison of Elastomeric Materials for Hydraulic Seal Durability under Reciprocating Conditions

Vishal Kumar and Muthu Elen *

Energy and Environment Directorate, Pacific Northwest National Laboratory, Richland-99354, Washington, USA.Vishal.Kumar@pnnl.gov
* Correspondence: Muthu.Elen@pnnl.gov

Analysis of Variance (ANOVA) Results for COF and Specific Wear Rate

Material E1

Table S1: ANOVA for Coefficient of Friction (COF)

Source of Variation	Sums of Squares SS	Degrees of freedom DF	Mean Squares MS	F calculated	F0.05	p-value
Between Load	0.05029	2	0.02514	180.84	3.55	1.209e-12
Between Speed	0.02596	2	0.01298	93.35	3.55	3.143e-10
Load × Speed (Interaction)	0.27782	4	0.06945	499.54	2.92	1.11e-16
Error (residual)	0.0025	18	0.00014			
Total	0.35657	26				

Table S2: ANOVA for Specific Wear Rate

Source of Variation	Sums of Squares SS	Degrees of freedom DF	Mean Squares MS	F calculated	F0.05	p-value
Between Load	0.97354	2	0.486	27.93	3.55456	3.028e-6
Between Speed	9.77049	2	4.885	280.36	3.55456	2.72e-14
Load × Speed (Interaction)	11.79638	4	2.942	169.24	2.92774	5.118e-14

Error (residual)	0.31365	18	0.0172			
Total	22.85406	26				

Material E2

Table S3: ANOVA for Coefficient of Friction (COF)

Source of Variation	Sums of Squares SS	Degrees of freedom DF	Mean Squares MS	F calculated	F0.05	p-value
Between Load	0.14485	2	0.07243	151.59	3.55456	3.028e-6
Between Speed	0.19455	2	0.09727	203.59558	3.55456	2.72e-14
Load × Speed (Interaction)	0.62368	4	0.15592	326.34337	2.92774	5.118e-14
Error (residual)	0.0086	18	0.00048			
Total	0.97168	26				

Table S4: ANOVA for Specific Wear Rate

Source of Variation	Sums of Squares SS	Degrees of freedom DF	Mean Squares MS	F calculated	F0.05	p-value
Between Load	20.07512	2	10.03756	5606.18222	3.55456	1.11e-16
Between Speed	43.50989	2	21.75494	12150.58287	3.55456	1.11e-16
Load × Speed (Interaction)	62.7782	4	15.69455	8765.72839	2.92774	1.11e-16
Error (residual)	0.03223	18	0.00179			
Total	126.39543	26				

Material E3

Table S5: ANOVA for Coefficient of Friction (COF)

Source of Variation	Sums of Squares SS	Degrees of freedom DF	Mean Squares MS	F calculated	F0.05	p-value
Between Load	1.81769	2	0.90884	436.63345	3.55456	5.551e-16
Between Speed	0.17696	2	0.08848	42.50712	3.55456	1.518e-7
Load × Speed (Interaction)	0.33176	4	0.08294	39.84609	2.92774	1.037e-8
Error (residual)	0.03747	18	0.00208			
Total	2.36387	26				

Table S6: ANOVA for Specific Wear Rate

Source of Variation	Sums of Squares SS	Degrees of freedom DF	Mean Squares MS	F calculated	F0.05	p-value
Between Load	222.98206	2	111.49103	33173.44402	3.55456	1.11e-16
Between Speed	114.40289	2	57.20144	17019.9243	3.55456	1.11e-16
Load × Speed (Interaction)	29.22695	4	7.30674	2174.07289	2.92774	1.11e-16
Error (residual)	0.0605	18	0.00336			
Total	366.67239	26				

Material E4

Table S7: ANOVA for Coefficient of Friction (COF)

Source of Variation	Sums of Squares SS	Degrees of freedom DF	Mean Squares MS	F calculated	F0.05	p-value
Between Load	93.75556	2	46.87778	0.97851	3.55456	0.395
Between Speed	95.77922	2	47.88961	0.99963	3.55456	0.3876
Load × Speed (Interaction)	189.42121	4	47.3553	0.98847	2.92774	0.4388
Error (residual)	862.33508	18	47.9075			
Total	1241.29107	26				

Table S8: ANOVA for Specific Wear Rate

Source of Variation	Sums of Squares SS	Degrees of freedom DF	Mean Squares MS	F calculated	F0.05	p-value
Between Load	1204.73776	2	602.36888	173760.25321	3.55456	1.11e-16
Between Speed	421.79449	2	210.89724	60835.74359	3.55456	1.11e-16
Load × Speed (Interaction)	1125.34742	4	281.33686	81154.86218	2.92774	1.11e-16
Error (residual)	0.0624	18	0.00347			
Total	2751.94207	26				

Material E5

Table S9: ANOVA for Coefficient of Friction (COF)

Source of Variation	Sums of Squares SS	Degrees of freedom DF	Mean Squares MS	F calculated	F0.05	p-value
Between Load	0.61521	2	0.3076	835.0365	3.55456	1.11e-16
Between Speed	0.09529	2	0.04765	129.34275	3.55456	2.087e-11
Load × Speed (Interaction)	0.03661	4	0.00915	24.84406	2.92774	4.043e-7
Error (residual)	0.00663	18	0.00037			
Total	0.75374	26				

Table S10: ANOVA for Specific Wear Rate

Source of Variation	Sums of Squares SS	Degrees of freedom DF	Mean Squares MS	F calculated	F0.05	p-value
Between Load	171.83045	2	85.91523	21964.25747	3.55456	1.11e-16
Between Speed	231.99176	2	115.99588	29654.38634	3.55456	1.11e-16
Load × Speed (Interaction)	32.10298	4	8.02574	2051.78438	2.92774	1.11e-16
Error (residual)	0.07041	18	0.00391			
Total	435.9956	26				
